# Effect of Froude Number on Submerged Gas Blowing Characteristics

**DOI:** 10.3390/ma14030627

**Published:** 2021-01-29

**Authors:** Jonas L. Svantesson, Mikael Ersson, Pär G. Jönsson

**Affiliations:** Department of Materials Science and Engineering, KTH Royal Institute of Technology, 114 28 Stockholm, Sweden; bergsman@kth.se (M.E.); parj@kth.se (P.G.J.)

**Keywords:** compressible flow, incompressible flow, IronArc, OpenFOAM, modified Froude number, submerged gas blowing, gas jetting

## Abstract

The flow behavior of gas in compressible and incompressible systems was investigated at an ambient temperature in an air–water system and at an operating process temperature in the IronArc system, using computational fluid dynamics. The simulation results were verified by experiments in the air–water system and established empirical equations to enable reliable predictions of the penetration length. The simulations in the air–water system were found to replicate the experimental behavior using both the incompressible and compressible models, with only small deviations of 7–8%. A lower requirement for the modified Froude number of the gas blowing to produce a jetting behavior was also found. For gas blowing below the required modified Froude number, the results illustrate that the gas will form large pulsating bubbles instead of a steady jet, which causes the empirical equation calculations to severely underpredict the penetration length. The lower modified Froude number limit was also found to be system dependent and to have an approximate value of 300 for the studied IronArc system. For submerged blowing applications, it was found that it is important to ensure sufficiently high modified Froude numbers of the gas blowing. Then, the gas penetration length will remain stable as a jet and it will be possible to predict the values using empirical equations.

## 1. Introduction

In metallurgical processes, such as the argon-oxygen decarburization (AOD) converter and ladle furnace, the use of submerged gas nozzles and tuyeres is a major part of the process design. Nozzles or tuyeres are used to inject gas below the surface of the molten metal to cause reactions and stirring. In other metallurgical processes, such as the electric arc furnace (EAF) and blast furnace, submerged oxy-fuel burners are used with combustible gas to provide heat in local regions.

In the AOD converter, oxygen is injected to react with and to reduce the amount of dissolved carbon in the steel. The blowing of oxygen is also often combined with inert gases such as argon and nitrogen to prevent the unnecessary oxidation of chromium, which is a valuable alloy in the steel. Additionally, gas injections are used to provide stirring to the process. Due to the extreme conditions in the melt, a mechanical stirring by impeller is hard to achieve, as the impeller will quickly wear down from the heat and reactions with the steel. By injecting gas into the melt, the bubbles which form will provide stirring by the drag they produce when rising to the surface [1].

Depending on the gas blowing parameters, the gas will either form discrete bubbles or a plume of coalescing bubbles. For use with low flow rates of gas, porous plugs or tuyeres are often used during bottom blowing in the ladle. These bubbles are mainly propelled by buoyancy and rise dependent on the ratio of the gas to melt density. For higher gas flows, side-blowing through a nozzle can be used to create a gas jet that propagates further into the melt. The gas is propelled into the melt with a high velocity and then rises due to buoyancy.

In most metallurgical processes, the gas is injected at ambient temperature or slightly below ambient temperature due to compression effects [2]. The contact with the hot molten metal leads to a rapid heating of the gas, which causes an expansion according to the natural gas law (Equation (1), where P is pressure, V is the volume, n is the amount of mole of gas, R is the gas constant, and T is the temperature). This expansion is partially counteracted by the increasing hydrostatic pressure from the weight of the molten steel above the nozzle (Equation (2) [3], where ρ is the density, g is the gravity, and h is the liquid height).

(1) P·V=n·R·T

(2) P=ρ· g· h 

It is very difficult to directly measure or study the gas penetration and plume behavior in metallurgical systems due to the high temperature and opaque nature of liquid metals. Instead, the gas behavior is commonly predicted by using a combination of water modelling and Computational Fluid Dynamics. Studies of the air–water system can be applied to the gas–metal system by scaling based on dimensionless numbers, such as the modified Froude number. The modified Froude number (N_Fr’_) is based on the ratio of inertial forces to buoyancy forces and is defined either as Equation (3) or Equation (4) [4].

(3) $$N_{Fr^{\prime }}=\frac{\rho_{g}\cdot u_{0}^{2}}{g\left(\rho_{l}-\rho_{g}\right)d_{0}}$$

(4) $$N_{Fr^{\prime }}=\frac{\rho_{g}\cdot Q_{g}^{2}}{\rho_{l}\cdot g\cdot d_{0}^{5}}$$

where the density of gas (ρ_g_) and liquid (ρ_l_), gravitational acceleration (g), and diameter of the inlet (d_0_) in the system is used together with the inlet velocity (u_0_) or the flow rate of the gas (Q_g_). The two definitions result in values that differ by a factor of π^2^/16, which is quite significant. For bottom blowing applications, there are other suggested formulations for the modified Froude number that are more appropriate when determining the plume behavior, as studied by Krishnapisharody and Irons [4]. For this study, the calculated penetration length is defined as a range between the values calculated using Equations (3) and (4).

The gas flow and dimensions of the inlet and reactor are scaled down to the water model to achieve a kinematic similarity between the water model and the industrial reactor. Based on verifying the physical experiments in the air–water system, a mathematical model can be set up in CFD and then scaled up to use the velocities and densities of the gas and liquids in the metallurgical system. This allows for accurate predictions of the bubble behavior in the metallurgical process without the need to measure it directly [5].

If the modified Froude number is equal in the model and the reactor, the flow behavior is expected to be similar. The flowrate for dynamic similarity can be determined by using Equation (5) [6]:

(5) $$Q_{m}=Q_{r}\cdot \lambda^{2.5}$$

where Q_m_ is the flowrate of the model, Q_r_ indicates the flowrate of the reactor, and λ is the geometric scaling factor between the reactor and the experimental setup. The latter is usually in the range of 1:3 to 1:10, which results in λ values of 1/3 to 1/10. To ensure equal modified Froude numbers, the diameter of the inlet (d_0_) does not follow the geometric scaling and is instead designed to satisfy Equation (6) [7].

(6) $$d_{0}=\sqrt[{5}]{\frac{\rho_{g}\cdot Q^{2}}{\rho_{l}\cdot g\cdot {\mathrm{Fr}}_{r}}}$$

Once an appropriate scaling is done, the model should produce results that are representative for the industrial size reactor. Based on this approach, several studies have been carried out to study the flow behavior in metallurgical processes [5,6,7,8,9,10,11,12].

Physical experiments to describe the jet trajectory of gas into liquid were first done by Themelis et al. to study the gas-blowing behavior in a copper converter [5]. The penetration length (L_p_) of side-blown gas jets in metallurgical applications has since then been studied using physical experiments in several different gas–liquid systems at room temperature, a few of which are listed in Table 1.

The experimental results from Hoefele and Brimacombe were used to establish an empirical equation for the penetration length (L_p_) of gas based on the modified Froude number in Equation (3), the diameter of the inlet, and the densities of the gas and liquid [8]. This empirical equation is widely used today and shown in Equation (7).

(7) $$L_{p}=10.7\;\cdot d_{0}{\left(N_{Fr^{\prime }}\right)}^{0.46}{\left(\frac{\rho_{g}}{\rho_{l}}\right)}^{0.35}\;$$

Other empirical equations to describe the penetration length (L_p_) have also been developed by Igwe et al. (Equation (8)), where the inlet pressure (P_1_) is used as a scaling factor [13]; by Ishibashi et. al. [14] (Equation (9)), who used the modified Froude number; and by Han et al. [15] (Equation (10), who developed one similar to the equation presented by Brimacombe et al.

(8) $$L_{p}=0.97P_{1}\cdot d_{0}+6.4\;$$

(9) $$L_{p}=3.7d_{0}\cdot N_{Fr^{\prime }}^{\frac{1}{3}}\;$$

(10) $$L_{p}=3.765{\left(\frac{\rho_{g}\cdot u_{g}^{2}}{\left(\rho_{l}-\rho_{g}\right)g\cdot d_{o}}\right)}^{0.35}$$

The behavior of a submerged gas jet in liquid is inherently unsteady and turbulent, which makes accurate assessment of the penetration length problematic. In previous studies, it has been found that the stability of the submerged jet is dependent on the nozzle diameter and geometry, as well as the modified Froude number [16]. Lin et al. also showed that increasing density ratios between the gas and liquid decreased the stability of the jet further [17].

In addition to the penetration length of the gas jet, the expansion angle of the gas jet has been found to be controlled by the ratio of the gas to liquid densities. Work by Oryall and Brimacombe found that the expansion angle of the injected gas into the ambient liquid is dependent on the liquid density rather than on the modified Froude number. This may cause significant back-expansion in metallurgical processes, which is not observed in water-model experiments. The expansion angle for air in mercury was found to be 155°, but in the air–water system the same angle was found to be only 20° [9]. This angle is most likely different depending on the application, which further complicates the prediction of penetration length in the actual process. Harby et al. also found that the expansion angle of gas was dependent on the nozzle diameter and flow rate of gas, but less so for low modified Froude numbers. Additionally, an increasing nozzle diameter and decreasing gas flow rate significantly increased the oscillation of the jet [18].

The entrainment of liquid droplets in a jet stream of air may also be significant when determining the penetration length and plume behavior of the gas injection. Depending on the modified Froude number and the jet velocity at the inlet, different amounts of ambient liquids will be entrained in the gas jet and increase the mass flux of the jet [18]. This will lead to increased modified Froude numbers if the injected gas has a higher apparent density than the pure gas due to the entrained liquid droplets.

The oscillations of the reactor, as present in the AOD process, are caused by the gas injection and has been studied thoroughly by Odenthal et al. and Fabritius et al., who found that reactor oscillations are prominent when the penetration lengths of the gas jets are too large [12,19]. However, the oscillations of the actual gas jet are expected to decrease with an increasing gas flow and modified Froude number [16]. As presented by Hoefele and Brimacombe, the oscillation frequency of the gas jet also decreases when blowing in a high-density liquid such as mercury as compared to water or a zinc-chloride solution [8]. It is expected that oscillations of the gas jet are caused when the gas flow is insufficient to form a steady stream of gas, instead the hydrostatic pressure of the liquid surrounding it will cause the gas to collapse and form discrete bubbles. This oscillation of the gas jet with high amplitudes is likely not present at high gas flow rates, as studied by Fabritius and Odenthal, instead some other property of the gas blowing is causing the reactor oscillations.

In the IronArc process, a plasma generator (PG) is used for injection of the reaction gas by side-blowing into the reactor. The PG superheats the gas by passing it through an electric arc before injecting it into the molten metal or slag. Since the gas is superheated, when it is injected into the reactor it will not expand in the same way as when blowing with cold gas. It will instead have a much higher velocity and lower density than gas injected at ambient temperature in established metallurgical processes. Depending on the power settings and gas flow in the PG, the temperature, velocity, and kinetic energy of the gas can be controlled [20].

All the previously listed equations for the penetration length and scaling assume isothermal systems and do not account for the surface tension between phases. The assumptions in these equations may be valid for the studied systems of molten metal and cold injected gas, but it is not certain that they are universal and cover the IronArc system of the molten slag and hot injected gas. The experimental studies were done in a large range of modified Froude numbers from 5–11,000, but a soft lower limit of 200–500 was also indicated by Brimacombe and Oryall for where the behavior changes from a bubbly flow to a jetting flow [8]. Below this limit the jet is extremely unstable and has large amplitudes in the oscillations due to the bubbles, which also makes measurement of the penetration length quite uncertain.

To include the effect of surface tension, work by Zhao and Irons [21] suggest that a Weber number criterion is more appropriate for prediction of the penetration length than the modified Froude number criterion as previously used. However, more recent work by Ma et. al. [22] used the Buckingham pi theorem to develop a new dimensionless formation for the penetration depth based on the modified Froude number combined with the Reynolds number. From the work by Ma et al., it was further confirmed that the widely used equation of the penetration length by Hoefele (Equation (7)) is well suited for high Fr’ numbers but not for low Fr’ numbers, where it severely underpredicts the penetration length.

The injection of hot gas through a PG may not produce the same behavior in terms of the penetration length of the gas jet as with a cold gas through a nozzle. This, in turn, will affect the bubble plume and stirring of the melt inside the reactor. Previous studies of the penetration length and mixing time in the IronArc reactor were performed by Bölke et al. with the assumption that scaling from a physical model in the air–water system using the previously presented equations appropriately reflected the flow behavior in the IronArc reactor [23].

The gas behavior of a hot gas entering a hot liquid is perhaps more like the water model than metallurgical systems using cold gas that will expand in the reactor. However, in the work by Odenthal et al., the compressibility and thermal expansion was included when the initial penetration of the gas jet was modelled. A good agreement was found with the empirical equation (Equation (7)) by Brimacombe et. al. at the working point of the AOD at a modified Froude number of 3268 [12].

Experiments in an air–water system as a scale replica of the IronArc reactor by Bölke et al. suggests that the empirical equation by Brimacombe and Oryall significantly underpredicts the penetration length for the IronArc reactor. However, those simulations were done with low modified Froude numbers from 2–90, which may be below the threshold for a jetting behavior [24]. At such conditions, the pulsing behavior of the jet would make accurate measurements of the actual penetration length impossible.

The current work aims to study the behavior of gas blowing in liquids for varying gas and liquid densities and surface tensions. An accurate modelling of the gas blowing behavior from a plasma generator is important to determine the viability of the IronArc reactor, due to the requirements on stirring and refractory. The lower limit on the modified Froude numbers for valid calculations using the empirical equation in Equation (7) will be investigated for the IronArc system.

## 2. Materials and Methods

The study was performed by CFD simulations using OpenFOAM v.7 (The OpenFOAM Foundation, London, UK) in Linux Mint 18.3 on an Intel Core i9-7940X (Intel Corporation, Santa Clara, CA, USA) with 14 cores and 32 GB of RAM, and in Ubuntu 18.04 (Canonical, London, UK) with a dual AMD EPYC 7301 (AMD, Santa Clara, CA, USA) with 16 cores each and 128 GB of RAM [25]. This was done to study how the penetration of gas into liquid is affected by the density ratio between the gas and liquid and to determine the lower limit for the modified Froude number for the jetting behavior. The accuracy of the established empirical equation for gas penetration presented in Equation (7) was also studied to see how well it predicts the flows in the IronArc process at high temperature and density ratios, as the empirical equation was not designed for such systems.

### 2.1. Simulation Domain and Meshing

**Figure 1:**
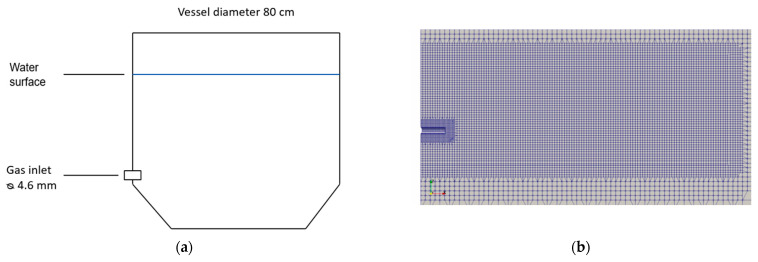
Sample mesh for the air–water system (**a**) and a sketch of the physical modeling system (**b**).

The simulation domain was constructed in Ansys SpaceClaim (Ansys Inc., Canonsburg, PA, USA) v.19.1 as a rectangular box with a side inlet. The domain was split along a symmetry line through the inlet to reduce the computational requirement for the simulations. For the simulations at room temperature the rectangular box was 1 m × 0.4 m × 0.75 m, with the liquid surface level patched in at 0.55 m. The inlet was circular with a diameter of 4.6 mm and extended 16 mm into the domain at a height of 98 mm above the bottom. For the plasma generator simulation, a larger geometry of 2 m × 1 m × 2 m was used, and the inlet size was changed to 30 mm in diameter, as is present on the 3 MW plasma generator used by ScanArc (Hofors, Sweden). The domain was split at the liquid surface level of 1.6 m to ensure that the patched liquid height was not affected by the meshing. A sample of the mesh in the refined area and a sketch of the physical modeling system are shown in Figure 1.

The domains were meshed using the cut-cell approach in Ansys^®^ Meshing v.19.1 with a refinement region close to the inlet and in the jetting area that extends into the domain. When using the cut-cell approach, all cells of the mesh are hex-sided cells, as such cubic cells do not experience the common skewness or aspect ratio errors as tetragonal mesh cells. For the mesh sensitivity analysis, three different meshes were constructed with decreasing element sizes in the inlet and refinement areas. The mesh was then converted to an OpenFOAM compatible mesh using the integrated fluent3DMeshToFoam command. This converted mesh is polyhedral to capture the curved surfaces, which introduces some skewness to the meshes. The air–water system mesh had a maximum skewness of 0.667 and the plasma generator mesh had a maximum skewness of 0.535. The mesh analysis was done according to the procedure established by Richardson and further developed by Celik et. al. and focused on the penetration length (L_p_) of the gas into the liquid, as measured through the nozzle centerline to the point where the fraction of gas is less than 10% [26,27]. The representative cell size was not calculated using the total number of cells in the domain, but rather set as the cell size in the refinement area. The GCI of the mesh was calculated using Equation (11) where e^21^ is the relative error between the fine and medium mesh and $r_{21}^{p}$ is the grid refinement factor from the medium to fine mesh using the apparent order of the calculation (p).

(11) $${\mathrm{GCI}}_{\mathrm{fine}}^{21}=\frac{1.25e_{a}^{21}}{r_{21}^{p}-1}$$

The mesh analysis was performed for the incompressible, isothermal simulations in the air–water system, as well as for the compressible simulations in the air–water system and in the PG-blowing system. The results from the mesh sensitivity analysis and studied mesh sizes are presented in Table 2 and Table 3.

The mesh sensitivity analysis shows a grid convergence of 2.4% in the incompressible simulations, 18% in the compressible simulation, and 0.04% in the IronArc system. For the compressible simulations, it was found that the mesh also affected the inlet pressure and, in turn, the gas density and the average inlet velocity, since the flow is pressure driven. The increasing velocity with mesh refinement exaggerates the difference in penetration length in the compressible simulations in the air–water system. In general, the incompressible simulations in the air–water system and the compressible simulations in the IronArc system are considered sufficiently mesh independent.

### 2.2. Solver Settings and Simulation Theory

The incompressible simulations were solved by using the InterFoam solver in OpenFOAM v.7, which uses the volume of fluid approach suggested by Hirt et al. to solve the continuity equation for two incompressible, isothermal, and immiscible fluids [25,28]. The continuity of the InterFoam solver is maintained by the constant-density continuity equation, as presented in Equation (12), which means that the net mass flux into a control volume is zero.

(12) $$\frac{\partial \left({\rho u}_{j}\right)}{\partial x_{j}}=0\;$$

The flow behavior within the InterFoam solver is calculated by using the momentum equation, as presented in Equation (13). It considers the influence of gravity (g), density (ρ), velocity (u), pressure (p), viscous (τ), and turbulent stress (τ_t_), as well as the source term including the surface tension between the phases.

(13) $$\frac{\partial \left({\rho u}_{i}\right)}{\partial t}+\frac{\partial }{\partial x_{j}}\left({\rho u}_{j}u_{i}\right)=-\frac{\partial p}{\partial x_{i}}+\frac{\partial }{\partial x_{j}}\left(\tau_{\mathrm{ij}}+\tau_{t}{}_{\mathrm{ij}}\right)+{\rho g}_{i}+f_{\sigma i}\;$$

The density in each computational cell in the system is calculated based on the fraction of each phase (α) according to Equation (14).

(14) $$\rho ={\alpha \rho }_{1}+\left(1-\alpha \right)\rho_{2}$$

Additionally, the interphase between the two phases is calculated by Equation (15).

(15) $$\frac{\partial \alpha }{\partial t}+\frac{\partial \left({\alpha u}_{j}\right)}{\partial x_{j}}=0\;$$

The divergence schemes used for the simulation are based on the Gauss interpolation from the cell centers to the face centers. This is combined with the upwind or van Leer discretization schemes, depending on the required accuracy for the specific property. The compressible simulations were solved by the compressible InterFoam solver in OpenFOAM 7, which also is based on the volume of fluid method. However, this solver also considers the compressibility effect on the fluids in the system when the pressure or temperature changes. To incorporate these effects in the calculation, the density of the fluids is variable and calculated using the equation of state for a perfect gas for the gasses and perfect liquid for the liquids, as described in Equation (16).

(16) $$\rho =\frac{p}{\frac{R}{M_{w}}T}$$

where ρ is the density, P is the pressure, T is temperature, R is a fluid constant, and M_w_ is the molecular weight. For all perfect gases, the fluid constant is 8.314 (J·mol^−1^·K^−1^) and for air the molecular weight is 28.9 (g·mol^−1^). For liquids, these values are harder to determine but, in most cases, also not as significant, as liquids behave more incompressible and experience lower temperature changes than gases. For the slag in the IronArc system, the R value is chosen as 3000 (J·mol^−1^·K^−1^), based on the depth charge tutorial case in OpenFOAM, and the molecular weight is 70 (g·mol^−1^) [25].

The use of turbulence models for the CFD modelling of the gas blowing processes has been reviewed by Ersson and Tilliander, where it is apparent that the k-ε model is most widely used for side-blowing modelling [29]. Turbulence models are based on the Navier–Stokers equation, which describe the flow of fluids. The velocity of the flow causes vortices due to shearing when obstructed by an obstacle or near a wall; these vortices feed on the swirl of smaller vortices. Those, in turn, feed on smaller vortices, down to the microscopic scale where they are dissipated by viscosity. To resolve these vortices in a simulation, the mesh would need to be on the same scale as the smallest vortices, which is not feasible for an advanced simulation. These vortices can instead be modelled using the Boussinesq approximation for turbulence, which utilizes the fact that a flowing liquid appears to have increasing viscosity as the turbulence increases [30]. By implementing a turbulent viscosity (eddy viscosity) to represent this phenomenon, the flow can be modelled in an averaged way using the RANS models (Reynolds-averaged Navier–Stokes). For a more detailed simulation, the LES (Large Eddy Simulation) approach can be used where the small vortices (eddies) are modelled using the Boussinesq approximation and the large eddies are properly resolved. This vastly increases the computational demand for the simulations since the mesh size must be sufficiently fine to resolve the large eddies.

A compromise of the simulation detail in between the RANS models and the LES models is found in the DES models (Detached Eddy Simulation). The DES models are intended to reduce the requirements on the mesh by implementing a LES model in the areas of the domain where the mesh is sufficiently fine to resolve the turbulent eddies and a RANS model (such as the k-ω or k-ε) in the areas with a coarser mesh. This gives a good combination of accuracy and efficiency for the simulations.

The turbulence for this work is solved using the kOmegaSSTDES turbulence model in OpenFOAM. The kOmegaSSTDES is a DES-type turbulence model that combines a LES behavior in the refined area close to the inlet and a k-ω SST model in the coarse region [31]. The k-ω SST model combines the good wall treatment used by the k-ω models with the good bulk flow treatment by the k-ε models by switching between them depending on the proximity to walls. For the kOmegaSSTDES model, the turbulent kinetic energy is calculated according to Equation (17), and the turbulent dissipation rate is calculated according to Equation (18).

(17) $$\frac{\partial \left(\rho k\right)}{\partial t}+\frac{\partial \left({\rho U}_{i}k\right)}{\partial x_{i}}={\tilde{P}}_{k}-\beta^{*}\rho k\omega +\frac{\partial }{\partial x_{i}}\left[\left(\mu +\sigma_{k}\mu_{t}\right)\frac{\partial k}{\partial x_{i}}\right]$$

(18) $$\frac{\partial \left(\rho \omega \right)}{\partial t}+\frac{\partial \left({\rho U}_{i}k\right)}{\partial x_{i}}={\alpha \rho S}^{2}-{\beta \rho \omega }^{2}+\frac{\partial }{\partial x_{i}}\left[\;\left(\mu +\sigma_{k}\mu_{t}\right)\frac{\partial \omega }{\partial x_{i}}\right]\;+2\left(1-F_{1}\right){\rho \sigma }_{w2}\frac{1}{\omega }\frac{\partial k}{\partial x_{i}}\frac{\partial \omega }{\partial x_{i}}\;$$

where ω is the turbulent dissipation rate, k is the turbulent kinetic energy, μ is the viscosity, U is the velocity, and F1 is the blending function that determines where the model should switch from the k-ε model to the k-ω model. The symbols α, β, and σ are used to denote different constants used to trim the model. Pk is a production limiter for the SST model to prevent excessive turbulence in the slow-moving regions of the simulation.

For this study, all simulations are done using the GaussSeidel solver for the pressure and the PBiCGStab solver for all other properties. PBiCGStab is a preconditioned bi-conjugate gradient solver for asymmetric IduMatrices using a run-time selectable preconditioner and the GaussSeidel solver is a run-time selected smoother.

### 2.3. Boundary Conditions

The simulations used a variable timestep to maintain a global courant number below 0.5. The initial timestep was set to 1 × 10^−7^ s and went as low as 5 × 10^−8^ for the simulations with the highest inlet velocities. All simulations were run to 2 s of flowtime, which allowed full development of the jets.

#### 2.3.1. Incompressible and Isothermal Simulation

Simulations of the air–water system were carried out as incompressible and isothermal simulations using the InterFoam solver and compared to water-modelling experiments as a verification. The boundary conditions used in the incompressible simulations are listed in Table 4.

The transport properties of the air and water used in the incompressible and isothermal simulations are listed in Table 5.

#### 2.3.2. Compressible Simulation

The compressible simulations were carried out using the compressible InterFoam solver for the air–water system and the IronArc system by using the settings for a plasma generator. The boundary conditions for the compressible simulations for the air–water system and the IronArc system are listed in Table 6 and Table 7.

The value of p_rgh is used to modify the inlet pressure for the parameter study in the IronArc system. The inlet pressure was started at 177 kPa, which is meant to produce the same gage pressure as in the compressible air–water system after compensating for the difference in hydrostatic pressure due to different liquids and bath height. The parameter study was done up to pressures of 300 kPa to investigate the inlet velocity and jet behavior with changing inlet pressure.

The air is defined as a perfect gas, which makes the density dependent on the pressure and temperature in the system. The liquids are also defined as perfect liquids, but the temperature dependence of the liquid density is insignificant due to very small changes in liquid temperature. The thermophysical properties for the air, water, and slag are listed in Table 8.

### 2.4. Density Variations

For a comparison with Equation (7), based on the modified Froude Number, several simulations were performed with different gases and liquids. The simulations were run with all combinations of the three gases—air, helium, and argon—and the three liquids—water, ZnCl-solution, and mercury. The simulations were run with the same boundary conditions as used in the incompressible simulations in the air–water system, as specified in Table 4 and using the medium mesh. The transport properties for the materials used in the simulations are listed in Table 9. Since the compressible and incompressible simulations for the air–water case produces very similar results, the incompressible model is used as it requires approximately a third of the computational time.

### 2.5. Validation

The simulations in the air–water system at ambient temperature were modelled to replicate the physical experiments done by Chanouian and Ahlin in an air–water system [32]. The experimental study found an average penetration length of 13.2 cm with a standard deviation of 2 cm for the air–water system at an inlet velocity of 240 m·s^−1^ through an inlet with a 4.6 mm diameter. This represents a modified Froude number of 1010–1642, depending on the equation used, which should be sufficient for a pure jetting behavior.

## 3. Results

### 3.1. Incompressible Air–Water System

**Figure 2:**
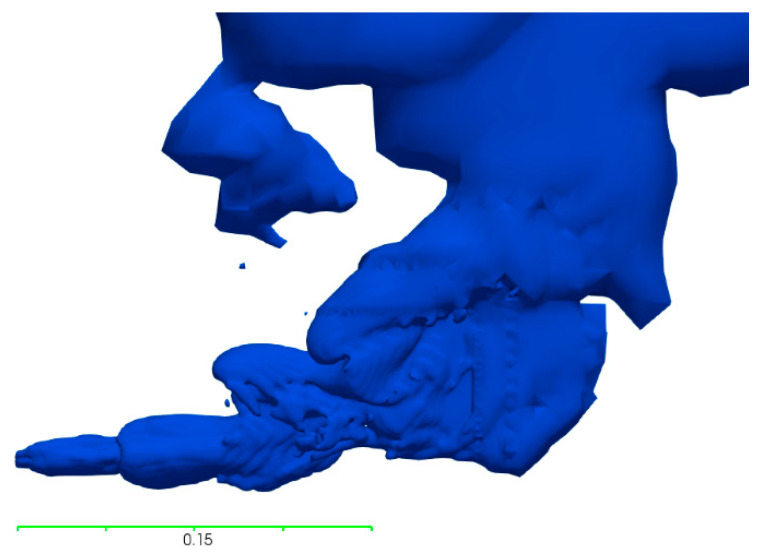
Gas plume of the incompressible simulation in the air–water system.

The incompressible simulations show good agreement with the water modelling and Equation (7) in terms of the gas penetration length, where the deviation of the simulated penetration length from the experimental value is 8.3% and the deviation from Equation (7) is less than 5%. In Table 10, the measured penetration lengths from the simulation and water modelling are shown with the calculated penetration length using the modified Froude number, based on both the flowrate and the velocity. The gas plume of the incompressible simulation is depicted in Figure 2 after two seconds of flowtime and looks very similar to the experimental gas plume presented by Chanouian and Ahlin [32].

Depending on the value of the liquid fraction used for the cut-off, the measured values for the penetration length differ slightly.

### 3.2. Compressible Air–Water System

**Figure 3:**
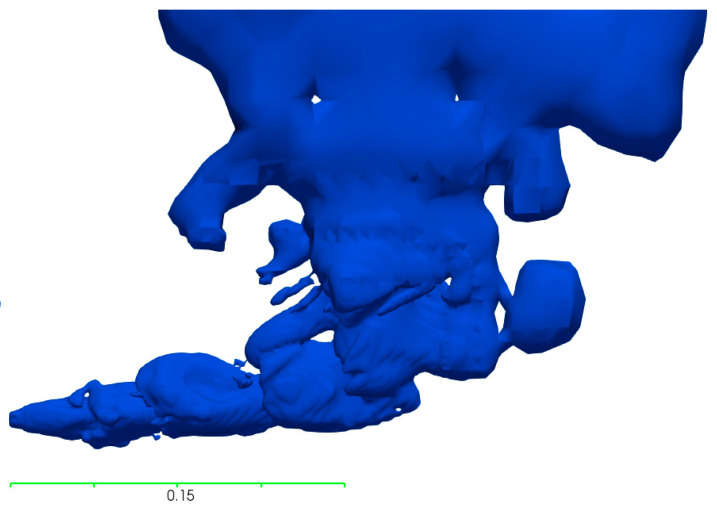
Gas plume of the compressible simulation in the air–water system.

In the compressible simulations, the air–water system produced penetration lengths in the same scale as the incompressible system at an ambient temperature. These results show similarities to both the water-modelling results and Equation (7), as can be seen in Table 10, with a deviation in penetration length of 7.8% from the experimental results and 10% from Equation (7). The flow behavior for the plume is very similar to the incompressible system, as can be seen when comparing the results in Figure 2 and Figure 3.

In the compressible system, the inlet velocity is determined by the pressure boundary condition rather than by an imposed velocity boundary condition. This causes fluctuations of the velocity at the inlet. Therefore, the velocity will not be exactly the same in the incompressible system and the compressible system. However, despite this, the similarities between the models are still clear. It should also be noted that the measured density of the gas at the inlet in the compressible air–water system is 1.36 kg·m^−3^ as compared to the set density of 1.2 kg·m^−3^ in the incompressible system. The change in density is not caused by differences in temperature, but rather because of the high pressure required to propel the flow as well as the hydrostatic pressure.

### 3.3. Density Variations

The results of the simulations in the incompressible system with gases and liquids of different densities at ambient temperature are listed in Table 11, together with the calculated penetration lengths from Equation (7) for each system. Since the simulations in the incompressible air–water system produced similar results and a better grid convergence index than the compressible simulations, the incompressible setup was used to simulate the varying gas–liquid combinations.

**Figure 4:**
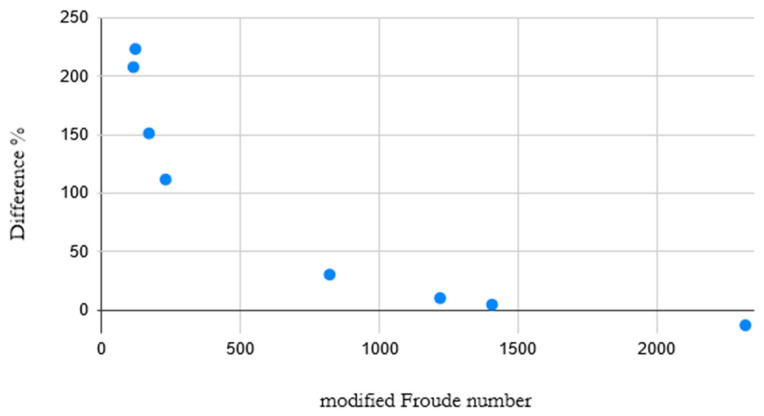
Plot of the modified Froude number vs. the difference between the simulation and empirical equation.

The deviation in penetration length of the simulated values as compared to the calculated values was calculated and plotted against the modified Froude Number and density ratio of each simulation. This comparison is shown in Figure 4.

### 3.4. The IronArc System

The gas blowing from the plasma generator in the IronArc system at high temperature was simulated at several different inlet pressures to study how the modified Froude number affected the flow behavior and penetration length. In general, the compressible simulations in the IronArc system show significantly larger gas expansion from the same applied inlet pressure as in the air–water system, due to the lower gas density at higher temperatures. In the center of the gas jet, where the temperature is close to 3000 K, the density of the gas is as low as 0.18 kg·m^−3^. 

**Figure 5:**
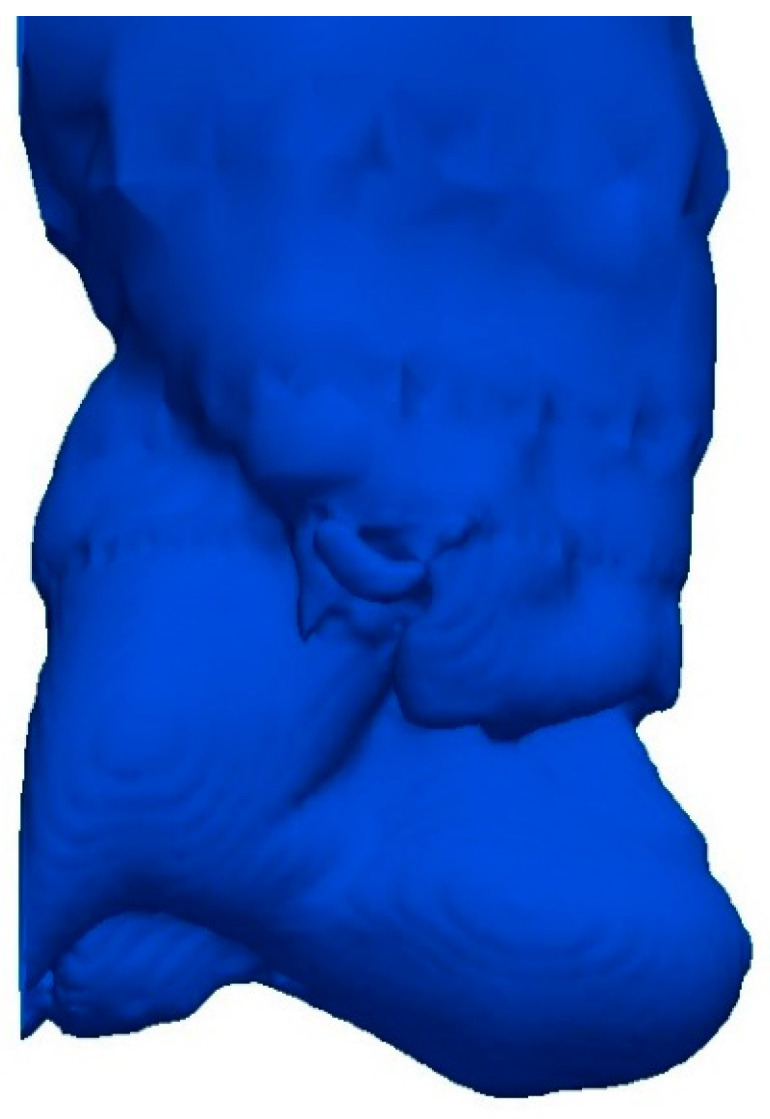
Gas blowing in the IronArc system with a pressure of 177 kPa at the inlet.

The different inlet pressures simulated in the IronArc system and their corresponding velocities, modified Froude numbers, penetration lengths, and calculated penetration lengths are presented in Table 12. The initial inlet pressure of 177 kPa produces an equal gage pressure in the inlet as in the air–water system after accounting for the hydrostatic pressure from the slag above the inlet. The behavior of that simulation is shown in Figure 5.

**Figure 6:**
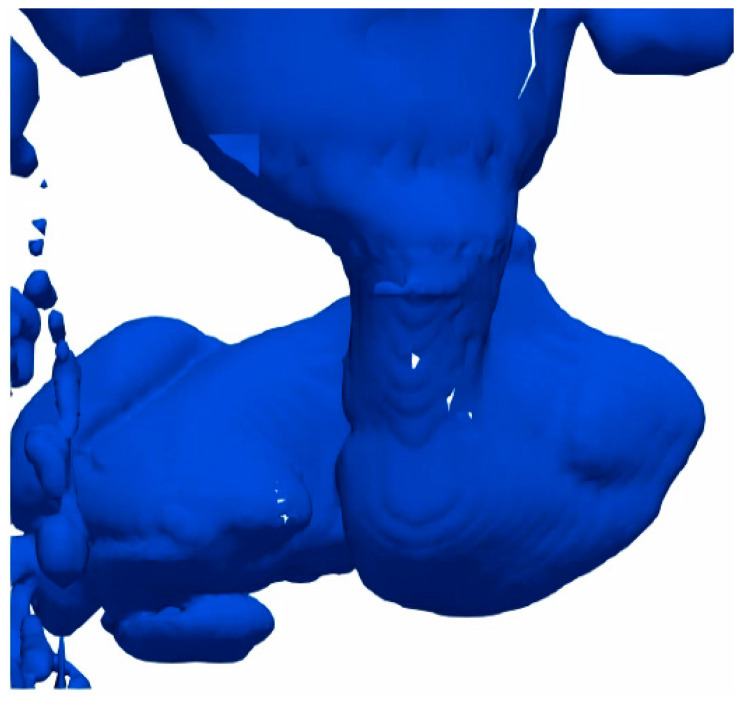
Gas blowing in the IronArc system with a pressure of 300 kPa at the inlet.

In Figure 5, it is apparent that the gas plume rises close to the wall despite the high measured and calculated penetration lengths. This is caused by the bubbling and pulsating gas behavior, due to a low modified Froude number. The modified Froude number presented in Table 12 is calculated using Equation (3) for the maximum and minimum velocity of each case based on the reported standard deviations. The calculated penetration lengths are therefore reported as a range based on the listed modified Froude numbers for each case. The simulated penetration lengths are significantly higher than the calculated penetration length for the low-pressure simulations with a difference of over 350%, but the difference decreases as the pressure used for the simulation increases. The difference in flow behavior between the low-pressure simulation and the high-pressure simulation can be seen when comparing the results in Figure 5 and Figure 6. The 300 kPa case, as presented in Figure 6, only exhibits a 5% difference in penetration length from Equation (7). At 300 kPa, the flow is still quite unstable and occasionally large bubbles form, as is indicated by the large standard deviation of the measured penetration length in Table 12. However, the majority of the injected gas flows away from the wall and rises in the domain, as opposed to the 177 kPa case in Figure 5 where almost all the gas rises close to the wall.

**Figure 7:**
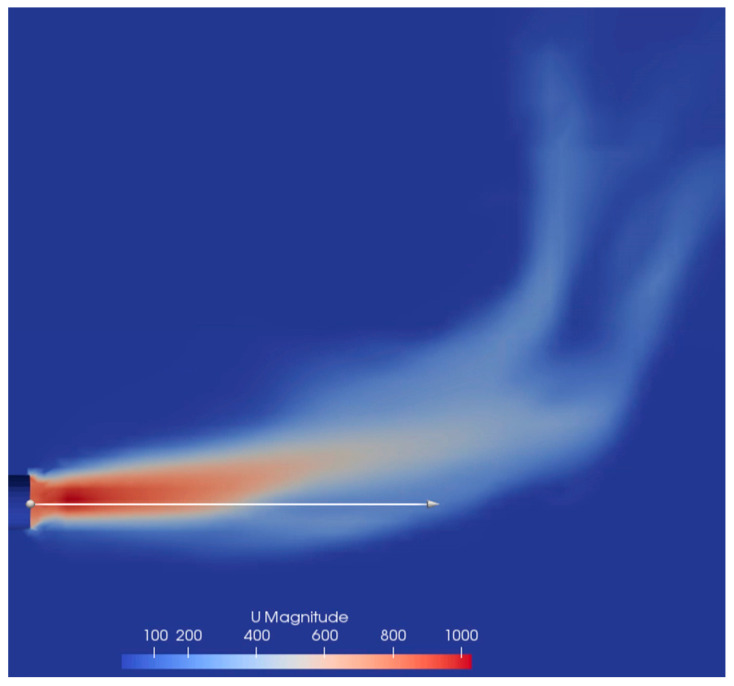
Velocity of the compressible simulation in the contour plot.

**Figure 8:**
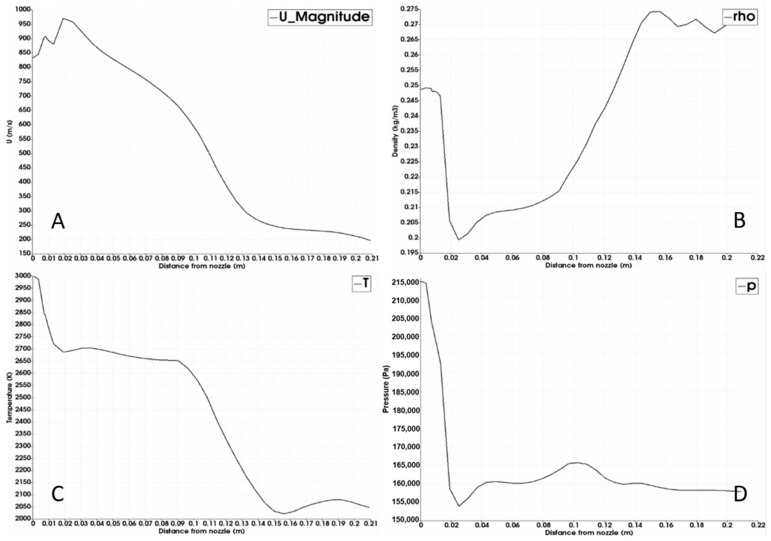
Properties of the compressible simulation: (**A**) velocity line; (**B**) density line; (**C**) temperature line; (**D**) pressure line.

The contour of the velocity in the compressible simulation is shown below in Figure 7 together with line plots along the indicated white line through the nozzle centerline of the velocity, density, temperature, and pressure in Figure 8.

It can be seen from Figure 7 and Figure 8 that the compressible gas reaches a maximum velocity close to the nozzle exit. At the same time there is a substantial drop in temperature and pressure (Figure 8C,D). It is likely that some of the jet energy is lost in terms of expansion and compression; however, it is difficult to quantify considering that the system is highly transient and that it also interacts with a liquid. The jet pressure (Figure 8D) drops from the nozzle pressure to the pressure of the surrounding liquid in about 0.02 m. At distances beyond 0.02 m, the pressure is close to the hydrostatic pressure when the surface is at the initial position. The density (Figure 8B) initially decreases as the temperature of the jet decreases in combination with the decrease in pressure. As the pressure stabilizes, there is an increase in pressure as the temperature continues to decrease.

## 4. Discussion

The gas blowing simulations in the incompressible air–water system fit reasonably well to the experimental work in the air–water system by Chanouian and Ahlin, which is used as validation for the study [32]. The simulations in the compressible air–water system are similar to the incompressible system, with the main difference being the higher density of the gas because of the high-pressure conditions. As shown in Table 10, Equation (7) predicts very similar penetration lengths for the incompressible and compressible simulations in the air–water system despite the slightly different velocities and densities of the systems. The increased density of the gas in the compressible system compensates for the slightly lower inlet velocity that sets almost equally predicted penetration lengths. However, the simulated penetration length of the incompressible and compressible models differs by 17%.

The mesh sensitivity analysis revealed that the mesh had a significant impact on the apparent pressure at the inlet in the compressible air–water system. This resulted in increasing velocities and measured penetration lengths with finer meshes, as can be seen in Table 2 and Table 3. This is thought to be caused by the poor resolution of the mesh in the areas above the inlet, which leads to differences in the calculation of the hydrostatic pressure. The large plume of gas above the inlet lowers the average density of the fluid, which leads to fluctuations in the hydrostatic pressure which, in turn, affect the stability and velocity of the injected gas jet. This phenomenon needs to be studied further to ensure that the simulations are sufficiently mesh independent to produce a reliable result. This behavior was also observed in the compressible simulations in the IronArc system, but to a lesser degree as the mesh dependence of these variables was only present when switching from the coarse mesh to the medium mesh, not from the medium mesh to the fine mesh.

Despite a 17% difference between the results from the incompressible and compressible simulations in the air–water system, the incompressible simulation setup was used for the study of the varying density ratios. This is motivated by that the incompressible simulation required approximately a third of the computational time as compared to the compressible simulation setup in the air–water system. Since the density variations are performed at isothermal conditions at ambient temperature, the impact of running the study in the incompressible system is expected to be small. The one property which is not considered when running the incompressible system is the changing density of the gas due to the hydrostatic pressure. As was observed in the air–water system, the pressure effect changed the density of the gas to 1.36 kg·m^−3^ from 1.2 kg·m^−3^ at the expense of velocity, which resulted in the very similar modified Froude numbers and mass flows of the simulations. For the systems with a ZnCl solution and mercury as the liquid the hydrostatic pressure will be significantly higher than in the air–water system, which may affect the density of a gas significantly.

From the varying density simulations, it was apparent that the density ratio between the liquid and gas affect the stability of the gas jet, even when equal inlet velocities are used. This behavior has also been observed previously by Lin et. al., who showed that increasing density ratios between the gas and liquid decreased the stability of the jet [17]. Additionally, increasing nozzle diameters and decreasing gas flow rates have also been shown to significantly increase the oscillation of the jet [18]. These three effects are connected since both increasing density ratios, increasing nozzle diameters, and decreasing gas flow rates will affect the modified Froude number of the gas blowing. A lower modified Froude number has previously been suspected to cause instabilities in the gas jet, as shown previously by Hoefele and Brimacombe [8]. If the modified Froude number is too low the gas plume will experience a bubbling and pulsing behavior rather than a jetting behavior. Bubbling and pulsing are analogous to an unstable jet and should be avoided to achieve good mixing and avoid interactions between the gas and the refractory wall. A clear example of a bubbling and pulsating jet can be seen in Figure 7 for the low-pressure simulation of the IronArc system. In this image, the rising plume can clearly be seen in contact with the wall of the domain since the modified Froude number of the gas blowing is too low.

The diagram of the gas blowing behavior presented by Brimacombe and Hoefele indicates that in order to ensure a steady jetting in the IronArc system, a modified Froude number of over 5000 is required. To reach such modified Froude numbers in the current geometry in the IronArc system, a velocity of approximately 5000 m·s^−1^ is required in the inlet, which is not feasible to simulate in this work. Instead, the lower end of the jetting region was investigated in the IronArc system by performing simulations of increasing pressures until a steady jetting behavior was found. However, there is no strict modified Froude number limit for when the gas plume shifts from a bubbling and pulsing to a jetting behavior. It is rather a soft transition that is system dependent and, for the IronArc system, starts at modified Froude numbers of roughly 300, below which the flow has significant bubbling and pulsing characteristics, and ends at modified Froude numbers around 5000, where the flow has pure jetting characteristics. As presented in Table 12, the penetration length for simulations with a low pressure are way underpredicted by Equation (7) since the pulsing of the gas reaches far out into the domain and skews the measurements. When the simulations are done with higher pressure, the predicted penetration length from Equation (7) and the simulated penetration length are much more similar. From these results it is determined that the lower limit for the modified Froude number for a jetting behavior in the IronArc system is 300. However, due to the large variations in velocity over time in the simulations, the jet is not always sufficiently stable and collapses, causing some pulsations even at the highest studied pressure.

The same behavior can be seen in the density variation simulations where it is apparent that the significantly different modified Froude numbers of the systems are correlated to the accuracy of Equation (7). As can be seen in the plot in Figure 6, the modified Froude number and the accuracy of Equation (7) is closely correlated for all the studied systems. In fact, the same limit that indicates when the blowing will exhibit a jetting behavior appears to be the lower limit for when Equation (7) will accurately predict the penetration length of the gas jet.

However, the lower limit of the modified Froude number is system dependent, as is evident in Figure 6, where simulations in different systems with similar modified Froude numbers show very different accuracies. Further work has to be done to determine if the system dependence is based in the density ratio as previously theorized or if another property, such as the viscosity of the liquid and/or gas, is the controlling factor. The jet behavior and accuracy of Equation (7) should also be studied for higher modified Froude numbers to see if the accuracy is stable after a certain modified Froude number or if it diverges at higher values.

This knowledge about the gas blowing allows us to improve the design of the inlets in many metallurgical processes to ensure that the gas injection is exhibiting a jetting behavior. To ensure a jetting behavior and a predictable penetration length, the modified Froude number of the gas injection should be increased. This can easily be done by reducing the diameter of the inlet where the plasma generator connects to the reactor to increase the modified Froude number of the gas blowing. When applying this change, calculations using Equation (7) shows that the actual penetration length will not increase when a smaller inlet is used, but rather decrease if used with the same inlet velocity. However, the higher modified Froude number indicates that the jet will be more stable and exhibit a less pulsating and bubbling behavior. A change in the inlet diameter while maintaining the volume flow rate through the nozzle would result in a higher velocity of the gas and an increased pressure requirement to propel the flow. This would further increase the penetration length and the modified Froude number, resulting in an even more stable jet. This theorized behavior of the gas jet should be studied further in future simulations to confirm the behavior.

A possible source of error in the simulations is that the penetration length from the simulations cannot be directly compared to Equation (7), since they are dependent on the bath height above the inlet that is not considered in the equation. The hydrostatic pressure is very important since it affects both the velocity (which is accounted for in the equation) but also the pulse amplitude and frequency. If the pulse amplitude and frequency changes, the average penetration length is hard to measure and the flow pattern will change in the domain.

For further studies, it would also be of interest to study how much of an effect the temperature of the gas has on the penetration length and gas plume behavior, as well as to determine if this needs to be considered when determining the required modified Froude number for jetting or if the changing density with increased temperature is sufficient.

## 5. Conclusions

This study aimed to investigate the gas blowing behavior from a plasma generator in the IronArc process to determine the penetration length of the gas jet and study how it could be simulated using incompressible and compressible simulations in OpenFOAM. It was found that gas blowing in an isothermal system at ambient temperature can be simulated with good accuracy by using both incompressible and compressible models. Compared to Equation (7), the incompressible simulations overpredicted the penetration length by 8.3% and the compressible simulations underpredicted the penetration by 7.8%. The difference in penetration length between the compressible and incompressible simulations is approximately 17%. However, the incompressible simulations require significantly lower computational effort than the compressible simulation for equal cases.

It was found that the modified Froude number of a gas blowing operation is intrinsically linked to the gas plume behavior and should be considered for metallurgical applications. If the modified Froude number of a gas-blowing application is not within or above the transition region, the gas plume will experience significant bubbling and pulsating behavior rather than jetting behavior. This may be problematic since the gas will produce a much longer maximum penetration length as compared to what is predicted by empirical equations, and also experience significantly more back-attack on the refractory wall. The back-attack on the refractory wall above the inlet will occur since the large bubbles that form in between the pulses simply rise straight up from the inlet and make contact with the back wall.

It was also found that the accuracy of Equation (7) for measuring the penetration length of the injected gas is dependent on the modified Froude number. For Equation (7) to be accurate, the injected gas must exhibit a jetting behavior, which requires a certain modified Froude number. For gas injection with modified Froude numbers below the limit, Equation (7) will severely underpredict the penetration length of the gas as the pulse amplitude can be very large. It was found that the lower limit for the modified Froude number to produce a partial jetting behavior in the IronArc process was approximately 300, which requires an inlet velocity of approximately 1300 m·s^−1^, depending on the gas density. However, even at this value, the gas jet occasionally collapses and comes in contact with the refractory wall.

This consideration should be implemented in the IronArc process to ensure a jetting behavior of the plasma generator gas. To increase the modified Froude number of the gas blowing the nozzle size should be decreased or the velocity of the gas should be increased. Such a change would reduce the amount of pulsations and bubbles from the inlet and allow for prediction of the penetration length of the gas with Equation (7).

## Figures and Tables

**Table 1:** Experimental studies of the penetration and plume behavior for varying gas and liquid densities.

Authors	Gas	Liquid
Hoefele and Brimacombe [8]	Air, Argon, Helium	Water, Zinc Chloride, Mercury
Oryall and Brimacombe [9]	Air	Mercury
Spesivtsev [10]	Air	Water, Mercury
Guthrie [11]	Air, Helium	Zinc Chloride
Odenthal [12]	Air	Water

**Table 2:** Mesh sensitivity analysis for the air–water system.

Mesh Size	Cell Size	Total Amount of Cells	Incompressible Lp (cm)	Compressible Lp (cm)	Compressible U (m·s^−1^)
Coarse Mesh	2 mm	163,000	7.6 ± 2.0	9.5 ± 2.1	204
Medium Mesh	1.4 mm	631,000	14.3 ± 2.5	12.2 ± 2.5	216
Fine Mesh	0.8 mm	1127,946	16.5 ± 3.6	14.5 ± 3.9	223
GCI			0.024	0.180	0.015

**Table 3:** Mesh sensitivity analysis for the IronArc system with a 77 kPa inlet overpressure.

Mesh Size	Cell Size	Total Amount of Cells	Lp (cm)	U (m·s^−1^)
Coarse Mesh	8 mm	140,000	9.7 ± 6.0	204 ± 187
Medium Mesh	6 mm	280,000	16.4 ± 8.7	393 ± 131
Fine Mesh	4 mm	991,000	17.4 ± 10.1	396 ± 165
GCI			0.004	2.7·10^−5^

**Table 4:** Boundary conditions used in the air–water system simulations.

Boundary	P_rgh	U	α-Water
Inlet	fixedFluxPressure	(240 0 0)	0
Outlet	fixedValue 0	pressureOutlet	0
Walls	fixedFluxPressure	noSlip	zeroGradient
Symmetry	symmetry	symmetry	symmetry

**Table 5:** Transport properties used in the incompressible simulations.

Boundary	Density (kg·m^−3^)	Viscosity (m^2^·s^−1^)	Surface Tension (N·m^−1^)
Water	1000	1.0 × 10^−6^	0.07
Air	1.200	1.5 × 10^−5^	

**Table 6:** Boundary conditions used in compressible simulation of the air–water system.

Boundary	U	P_rgh	P	T	α-Liquid
Internal	-	101,325	101,325	300	-
Inlet	pressureInlet	prghTotalPressure 150 kPa	internalField	300	inletOutlet 0
Outlet	pressureOutlet	prghPressure 100 kPa	internalField	300	fixedValue 0
Symmetry	Symmetry	Symmetry	Symmetry	Symmetry	Symmetry
Wall	noSlip	fixedFluxPressure	internalField	fixedValue	zeroGradient

**Table 7:** Boundary conditions used in compressible simulation of the IronArc system.

Boundary	U	P_rgh	P	T	α-Liquid
Internal	-	101,325	101,325	1700	-
Inlet	pressureInlet	prghTotalPressure 177kPa	internalField	3000	inletOutlet 0
Outlet	pressureOutlet	prghPressure 100kPa	internalField	1700	fixedValue 0
Symmetry	Symmetry	Symmetry	Symmetry	Symmetry	Symmetry
Wall	noSlip	fixedFluxPressure	internalField	fixedValue	zeroGradient

**Table 8:** Thermophysical properties for the compressible simulations.

Material	Molecular Weight (g·mol^−1^)	Specific Heat Cp (J·kg^−1^·K^−1^)	Dynamic Viscosity (Pa·s)	Pr Number	Base Density ρ_0_ (kg·m^−3^)	Surface Tension (N·m^−1^)
Air	29	1005	1.8 × 10^−5^	0.7	-	
Water	18	4191	3.6 × 10^−4^	2.3	1000	0.07
Slag	70	2000	1.0 × 10^−2^	10	3500	0.50

**Table 9:** Material properties used in the density variation simulations.

Material	Specific Heat, Cp (J·kg^−1^·K^−1^)	Kinematic Viscosity, ν (m^2^·s^−1^)	Base Density, ρ_0_ (kg·m^−3^)	Surface Tension (N·m^−1^)
Air	1005	1.5 × 10^−5^	1.2	-
Helium	5200	1.0 × 10^−4^	0.18	-
Argon	520	1.2 × 10^−6^	1.78	-
Water	4191	1.0 × 10^−6^	1000	0.07
ZnCl	1840	6.3 × 10^−6^	1900	0.07
Mercury	139	1.17 × 10^−6^	13600	0.47

**Table 10:** Measured and calculated penetration lengths from the compressible simulations in the air–water system.

Case	Velocity (m·s^−1^)	Density (kg·m^−3^)	Modified Froude Number	$\mathrm{Simulation}\;L_{p}\;(\mathrm{cm})$	Equation $L_{p}\;(\mathrm{cm})$	$\mathrm{Experimental}\;L_{p}\;(\mathrm{cm})$
Compressible	216 ± 2.9	1.36	865–1406	12.2 ± 2.5	11.0–13.5	13.2 ± 2.0
Incompressible	240	1.20	942–1531	14.3 ± 2.5	10.9–13.6	13.2 ± 2.0

**Table 11:** Results from the density variation simulations.

System	Modified Froude Number	Density Ratio: Liquid/Gas	Simulated $L_{p}\;(\mathrm{cm})$	Calculated $L_{p}\;(\mathrm{cm})$
Air + Water	865–1406	1000/1.2 = 833	14.3 ± 2.5	10.9–13.6
Air + ZnCl	506–821	1900/1.2 = 1583	10.5 ± 2.6	6.42–8.02
Air + Mercury	70–114	13,600/1.2 = 11,333	5.02 ± 2.1	1.30–1.63
Helium + Water	141–230	1000/0.177 = 5649	6.06 ± 2.3	2.29–2.86
Helium + ZnCl	74–121	1900/0.177 = 10,734	5.50 ± 2.3	1.36–1.70
Helium + Mercury	10–16	13,600/0.177 = 76,836	2.49 ± 1.4	0.27–0.34
Argon + Water	1426–2319	1000/1.78 = 561	16.2 ± 2.9	14.9–18.6
Argon + ZnCl	750–1219	1900/1.78 = 1067	12.2 ± 2.3	8.83–11.1
Argon + Mercury	104–170	13,600/1.78 = 7640	5.73 ± 2.4	1.79–2.24

**Table 12:** Simulated cases in the IronArc system at different inlet pressures.

Inlet Pressure	Gas Density	Simulated Velocity (m·s^−1^)	Modified Froude Number	$\mathrm{Simulated}\;L_{p}\;(\mathrm{cm})$	$\mathrm{Calculated}\;L_{p}\;(\mathrm{cm})$
177 kPa	0.188	393 ± 131	17–83	16.4 ± 8.7	4.4 ± 0.5
200 kPa	0.196	516 ± 165	23–88	20.3 ± 11.2	6.3 ± 1.8
225 kPa	0.208	636 ± 196	38–139	21.9 ± 11.4	7.0 ± 2.3
250 kPa	0.216	716 ± 248	41–176	16.8 ± 12.2	8.6 ± 2.8
300 kPa	0.226	963 ± 279	98–323	16.6 ± 16.2	12.6 ± 3.4

## Data Availability

Data is contained within the article.
